# The repositioned drugs disulfiram/diethyldithiocarbamate combined to benznidazole: Searching for Chagas disease selective therapy, preventing toxicity and drug resistance

**DOI:** 10.3389/fcimb.2022.926699

**Published:** 2022-07-29

**Authors:** Juliana Almeida-Silva, Diego Silva Menezes, Juan Mateus Pereira Fernandes, Márcio Cerqueira Almeida, Deyvison Rhuan Vasco-dos-Santos, Roberto Magalhães Saraiva, Alessandra Lifsitch Viçosa, Sandra Aurora Chavez Perez, Sônia Gumes Andrade, Ana Márcia Suarez-Fontes, Marcos André Vannier-Santos

**Affiliations:** ^1^ Innovations in Therapies, Education and Bioproducts Laboratory, Oswaldo Cruz Institute, Oswaldo Cruz Foundation, Rio de Janeiro, RJ, Brazil; ^2^ Parasite Biology Laboratory, Gonçalo Moniz Institute, Oswaldo Cruz Foundation, Salvador, BA, Brazil; ^3^ Laboratory of Clinical Research on Chagas Disease, Evandro Chagas Infectious Disease Institute, Oswaldo Cruz Foundation, Rio de Janeiro, RJ, Brazil; ^4^ Experimental Pharmacotechnics Laboratory, Department of Galenic Innovation, Institute of Drug Technology - Farmanguinhos, Oswaldo Cruz Foundation, Rio de Janeiro, RJ, Brazil; ^5^ Project Management Technical Assistance, Institute of Drug Technology - Farmanguinhos, Oswaldo Cruz Foundation, Rio de Janeiro, RJ, Brazil; ^6^ Experimental Chagas Disease Laboratory, Gonçalo Moniz Institute, Oswaldo Cruz Foundation, Salvador, BA, Brazil

**Keywords:** *Trypanosoma cruzi*, disulfiram, drug combination, repositioning, Chagas disease, chemotherapy, Diethyldithiocarbamate

## Abstract

Chagas disease (CD) affects at least 6 million people in 21 South American countries besides several thousand in other nations all over the world. It is estimated that at least 14,000 people die every year of CD. Since vaccines are not available, chemotherapy remains of pivotal relevance. About 30% of the treated patients cannot complete the therapy because of severe adverse reactions. Thus, the search for novel drugs is required. Here we tested the benznidazole (BZ) combination with the repositioned drug disulfiram (DSF) and its derivative diethyldithiocarbamate (DETC) upon *Trypanosoma cruzi in vitro* and *in vivo*. DETC-BZ combination was synergistic diminishing epimastigote proliferation and enhancing selective indexes up to over 10-fold. DETC was effective upon amastigotes of the BZ- partially resistant Y and the BZ-resistant Colombiana strains. The combination reduced proliferation even using low concentrations (e.g., 2.5 µM). Scanning electron microscopy revealed membrane discontinuities and cell body volume reduction. Transmission electron microscopy revealed remarkable enlargement of endoplasmic reticulum cisternae besides, dilated mitochondria with decreased electron density and disorganized kinetoplast DNA. At advanced stages, the cytoplasm vacuolation apparently impaired compartmentation. The fluorescent probe H_2_-DCFDA indicates the increased production of reactive oxygen species associated with enhanced lipid peroxidation in parasites incubated with DETC. The biochemical measurement indicates the downmodulation of thiol expression. DETC inhibited superoxide dismutase activity on parasites was more pronounced than in infected mice. In order to approach the DETC effects on intracellular infection, peritoneal macrophages were infected with Colombiana trypomastigotes. DETC addition diminished parasite numbers and the DETC-BZ combination was effective, despite the low concentrations used. In the murine infection, the combination significantly enhanced animal survival, decreasing parasitemia over BZ. Histopathology revealed that low doses of BZ-treated animals presented myocardial amastigote, not observed in combination-treated animals. The picrosirius collagen staining showed reduced myocardial fibrosis. Aminotransferase de aspartate, Aminotransferase de alanine, Creatine kinase, and urea plasma levels demonstrated that the combination was non-toxic. As DSF and DETC can reduce the toxicity of other drugs and resistance phenotypes, such a combination may be safe and effective.

## Introduction

At least 6-7 million people have Chagas disease (CD), mostly in Latin America (WHO - World Health Organization, 2021), where over 10% of the population is at risk of infection (Pérez-Molina and Molina, 2018). There are at least 4.6 million infected people in Brazil, which can reach 1.5% of the Brazilian population. In addition, about 70 million are at risk of infection by *Trypanosoma cruzi* (Dias et al., 2016). The parasitosis, also known as American trypanosomiasis, is already considered a public health problem on a global scale (Franco-Paredes et al., 2009; Coura and Viñas, 2010; Parker and Sethi, 2011).

CD causes economic losses in excess of U$1.2 billion/year to endemic countries in South America, in addition to more than $7 billion/year at global levels (Lee et al., 2013), including treatment and loss of productivity, not including the losses caused by infections by tourists and emigrants to North America, Europe, and Asia (Coura and Viñas, 2010) coming from South and Central America. Therefore, it can be inferred that effective drugs, besides promoting the quality of life of patients and their families, can provide considerable socioeconomic benefit.

Since CD discovery by the Brazilian researcher Carlos Chagas over a century ago, the disease is intensely studied, but only two drugs, benznidazole (BZ) and nifurtimox (NFX), are employed in CD treatment. However, BZ side effects lead to therapy discontinuation from approximately 30% of the cases up to eventually reaching 50% of the patients (Guggenbühl Noller et al., 2020). The option is NFX but a recent study (Crespillo-Andújar et al., 2018) reported that the use of NFX in patients who had been discontinued from BZ treatment still led to more than 12% of elevated toxicity, forcing physicians to permanently discontinue treatment. Therefore, there is pressing demand for the development of new drugs or therapeutic regimens for CD.

Different chemotherapy targets have been approached during the last decades (Duschak, 2011; Duschak, 2016; Beltran-Hortelano et al., 2017; Duschak, 2019; Beltran-Hortelano et al., 2022), and much was learned about the biochemistry and cell biology of *T. cruzi*, but new agents are still not in clinical use. Drug combinations may be promising for allowing dosing reduction (Bustamante et al., 2014), hampering resistance selection (Hill and Cowen, 2015), and enhancing selectivity (Zimmermann et al., 2007; Lehár et al., 2009a; Lehár et al., 2009b). In addition, drug combinations can promote effectivity of repositioning (Sun et al., 2016).

The use of repositioned drugs (approved by the FDA), with well-established data on bioavailability, safety, etc., allows accelerating drug development, significantly increasing the percentage of success, but reducing their costs (Zheng et al., 2018). Such innovations can be of great value in the therapy of neglected diseases, highly prevalent in South America, caused by parasitic protozoa (Müller and Hemphill, 2016), including CD (Palos et al., 2017).

The repositioning of a low-cost drug such as Disulfiram (DSF, Antabuse^®^) can be considered a “salvation” for global health care (Cvek, 2012). DSF, a drug used for the therapy of alcoholism, is widely used and well tolerated in humans (Jørgensen et al., 2011; Sinclair et al., 2016) and is even considered less toxic than aspirin (Gessner and Gessner, 1992) and trials employing 200-250 mg/d daily or 800 mg/twice a week are regularly performed, with no reports of adverse effects (Sinclair et al., 2016). The DSF first derivative sodium diethyldithiocarbamate (DETC), also known as imuthiol, has been successfully used as an immunostimulant in HIV patients, reducing opportunistic infections (Hersh et al., 1991). DSF is used for different purposes (e.g. Kona et al., 2011), such as cancer therapy (Cvek, 2012; Meraz-Torres et al., 2020; Kannappan et al., 2021; Lu et al., 2021; Lu et al., 2022) and chemoprevention (Askgaard et al., 2014; Yang et al., 2015; Harrington et al., 2020). The DSF and/or DETC combination can enhance antitumoral activities of drugs such as cisplatin (O’Brien et al., 2012; Nechushtan et al., 2015), but diminish adverse reactions (Wysor et al., 1982; Elliott et al., 1983; Bodenner et al., 1986; Roemeling et al., 1986). In addition, DSF can overcome resistance, *via* different mechanisms (Schmidtova et al., 2019; Yang et al., 2019). The data presented here indicate that the BZ-DSF combination may comprise a promising alternative for CD therapy.

## Materials and methods

### Drugs

BZ (Nortec Química, Rio de Janeiro, Brazil) and DSF (Corden Pharma Bergamo S.p.A) were provided by Farmanguinhos (Fiocruz, Rio de Janeiro) and DETC was purchased from Sigma-Aldrich. Drugs were dissolved in dimethyl sulfoxide (DMSO) and stored at -20°C until use.

### Parasites and mammalian cells

*T. cruzi* Y and the Colombiana strains epimastigote forms were maintained in LIT (Liver Infusion Trypticase) medium, supplemented with 10% fetal bovine serum (FBS), 100 µg/mL penicillin, and streptomycin at 25°C (dos Anjos et al., 2016). Cultures were harvested at the exponential growth phase. Then 5x10^5^ parasites were incubated in the presence of the isolated compounds. Trypomastigote forms were obtained by cardiac puncture, at the peak of parasitemia of infected Swiss Webster mice (Sueth-Santiago et al., 2016) and maintained through co-culture with epithelial cells, VERO, previously in Dulbecco’s modified Eagle’s medium (DMEM) supplemented with 10% FBS, at 37°C, 5% CO_2_, as well as the amastigote form obtained after 8 days of cell infection (Monteiro et al., 2001). Axenic amastigotes were obtained from cell cultures of trypomastigotes in BHT medium incubated at 28°C, being collected after three passages of 56-64 h, in an initial concentration between 5x10^6^ cells/mL (Engel et al., 1987). Macrophages from BALB/c mice were collected by peritoneal lavage in Hank’s balanced solution, seeded in 24-well plates or bottles (Falcon, New Jersey, USA), and kept at 37°C in atmosphere of 5% CO_2_ in DMEM medium supplemented with 10% FBS.

### Parasite-host interaction

Assays were performed using a parasite:cell ratio of 10:1, and infection quantification was performed by direct counting cultured cells Giemsa (Laborklin)-stained coverslips under light microscopy, approximately 1000 cells per coverslip. Association indices (AI) were obtained by multiplying the percentage of infected macrophages by the average number of parasites per host cell, as previously described (Martiny et al., 1996).

### *In vitro* evaluation of trypanocidal activity and cytotoxicity

*T. cruzi* epimastigotes (10^7^ cells/mL) incubated with DETC and BZ, to determine IC_50_ values each drug, at 24 h at 28°C, determined by the Alamar Blue assay at 570 nm and 600 nm. For the combinations, both for trypanocidal activity and for the selectivity index (10^7^ Swiss Webster mouse peritoneal macrophages/mL), six fixed doses were prepared based on the IC_50_ value of the isolated drugs, in the proportions 5:0, 4:1, 3:2, 2:3, 1:4 and 0:5 (Fivelman et al., 2004). Concentration-response curves were plotted and the IC_50_ and CC_50_ values of the compounds (inhibition and cytotoxicity, respectively), alone or in combination, were calculated using GraphPad Prism, 7.

### ROS detection

ROS were detected using the H_2_-DCFDA probe using a confocal microscope Fluoview 1000, Olympus.

### Lipid peroxidation

Lipid peroxidation was determined by the production of thiobarbituric acid (TBA) reactive substances (TBARS), by parasites incubated or not in the presence of the compounds for 24 h. Subsequently, the cells were centrifuged three times in phosphate-buffered saline (PBS). After washing, the parasites were resuspended in 200 μL PBS and 200 μL TBA at a final concentration of 1%. After homogenization, the material was incubated at 99°C for a period of 3 h and measured in a spectrophotometer at 532 nm (Menezes et al., 2006).

### Thiol group measurements

The determination of the concentration of low molecular weight thiols was carried out using 5,5′-dithiobis (2-nitrobenzoic acid) (DTNB) and methanol assay after protein removal with 10% trichloro acetic acid. Subsequently, the supernatant was read in a spectrophotometer at 412 nm (Hitachi U-1100), as previously described (Sedlak and Lindsay, 1968).

### Dosage of superoxide dismutase

Parasite samples untreated and treated with DETC alone and in combination with BZ, for 1 and 24 hours, were evaluated using a colorimetric method for superoxide dismutase (SOD) measurement (Sigma-Aldrich Kit-WST- SOD Assay), which is based on the generation of the radial superoxide from the xanthine-xanthine oxidase (XOD) system, where the superoxide reacts with the sample and converts the tetrazolium salt to formazan. After reactions, the measurements spectrophotometrically performed in a VersaMax at 440 nm (Moukdar et al., 2009).

### Electron microscopy

Parasites were washed with PBS and fixed in Karnovsky for 24 h, at 4°C. Then, samples were post-fixed in 1% osmium tetroxide, 5 mM calcium chloride, and 0.8% potassium ferricyanide in 0.1M sodium cacodylate buffer, protected from light for 40 min at room temperature. For scanning, electron microscopy (SEM) samples were dehydrated in ethanol series, critical point-dried, mounted on stubs, gold-metalized, and observed in a JEOL 5310 scanning electron microscope. For transmission, electron microscopy (TEM) samples were dehydrated in acetone and embedded in Polybed epoxy resin (Polysciences, Inc). After 72 h at 60°C, the samples were sectioned on an ultramicrotome (Reichert, Leica), using a diamond knife (Diatome, Hatfield, PA), and the sections were collected on 300 mesh copper grids and counterstained with 3% lead citrate and 5% uranyl acetate in water. Samples were observed in a transmission electron microscope Zeiss EM 109 at 80kV, as previously described (Vannier-Santos and Lins, 2001).

### *In vivo* infection

Swiss Webster mice were infected with Y or Colombian strain, intraperitoneally (i.p.), with 10^4^ bloodstream trypomastigotes. The infected animals were divided into the following groups (15 animals per group): positive control (BZ); negative control (PBS + 1% Kolliphor); DETC + BZ combination; DSF + BZ. The therapy was initiated 5 days from the beginning of the parasitemia and carried out for 30/60 days, administered through the intragastric route (Salomão et al., 2010). Parasitemia was verified by direct microscopic, slide analysis of parasites in 5 μL of blood, and mortality rates were checked daily/weekly up to 30 days post-treatment.

### Systemic toxicity

Thirty days after the end of the treatment, uninfected mice blood was collected through the brachial plexus and stored at -80°C for biochemical analysis of the enzymes: urea, for renal monitoring, total creatine kinase (CK), for evaluation of cardiac or skeletal musculature lesions, alanine aminotransferase (ALT) and aspartate aminotransferase (AST), assessment of liver damage, all being determined in whole blood by the Reflotron^®^ reactive test strip system (Roche Diagnostics, F Hoffmann-La Roche Ltd, Basel, Switzerland) using reflectance photometry (Salomão et al., 2010).

### Histopathology

Tissue samples were fixed in formaldehyde solution pH 7.2, embedded in paraffin, and 5 µm-thick sections were stained with hematoxylin and eosin (H&E) and picrosirius red to stain collagen fibers (Andrade et al., 1994).

### Statistical analysis

The data obtained are representative of at least three independent experiments carried out in triplicate. Statistically significant differences were analyzed using the ANOVA test and Tukey or Dunn post-tests with *p* < 0.05, using GraphPad Prism, 7.

## Results

**Figure 1:**
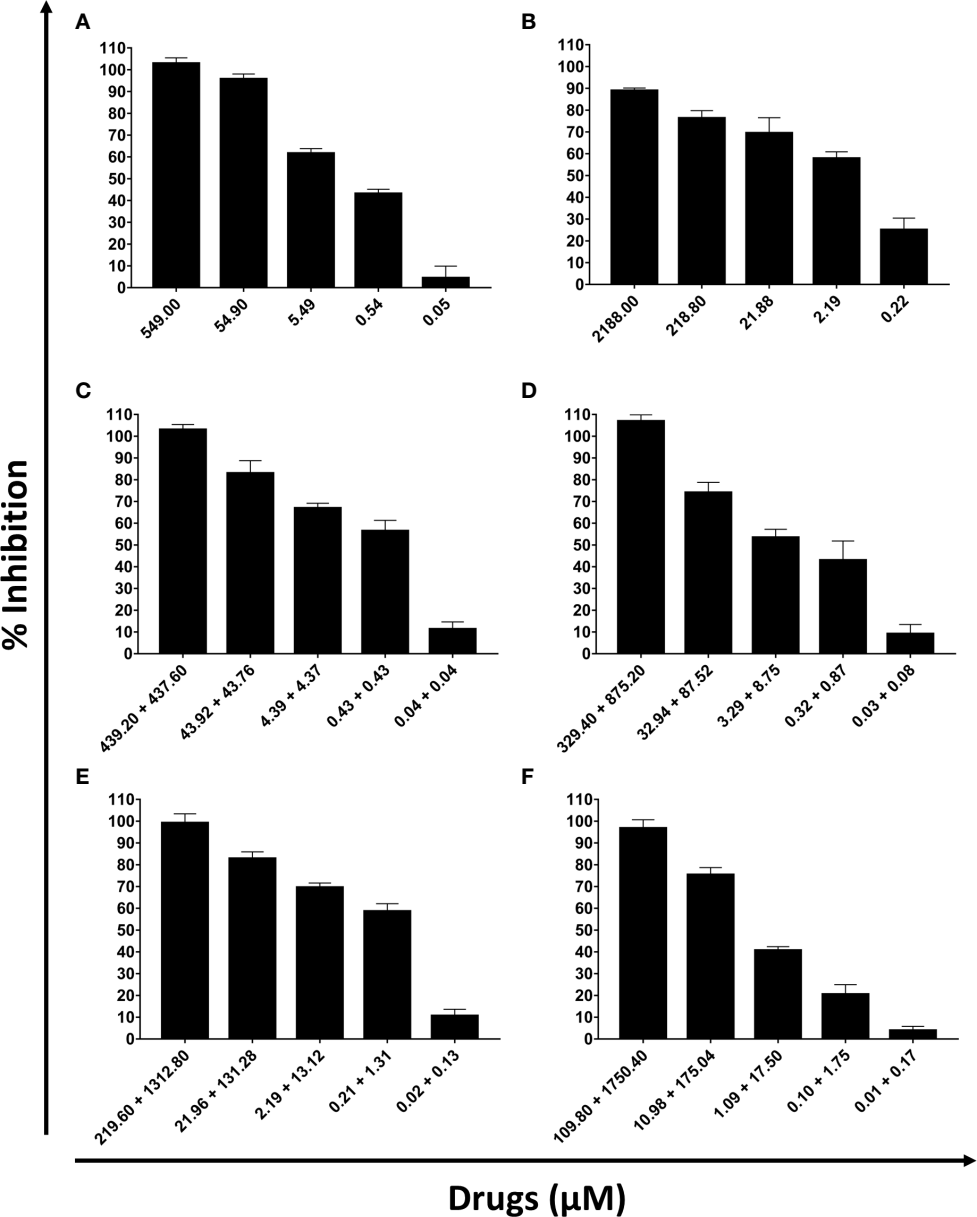
Evaluation of the activity of sodium diethyldithiocarbamate (DETC) and benznidazole (BZ) in the *in vitro* proliferation of *Trypanosoma cruzi* epimastigote forms (10^7^ parasites/mL, Y strain). The proportions of the combinations were: 5 x IC_50_ DETC **(A)**; 5 x IC_50_ BZ **(B)**, 4 x IC_50_ DETC + IC_50_ BZ **(C)**, 3 x IC_50_ DETC + 2 x IC_50_ BZ **(D)**, 2 x IC_50_ DETC + 3 x IC_50_ BZ **(E)** and IC_50_ DETC + 4 x IC_50_ BZ **(F)**. After 24h incubation, the inhibitory effects were determined using Alamar Blue.

**Figure 2:**
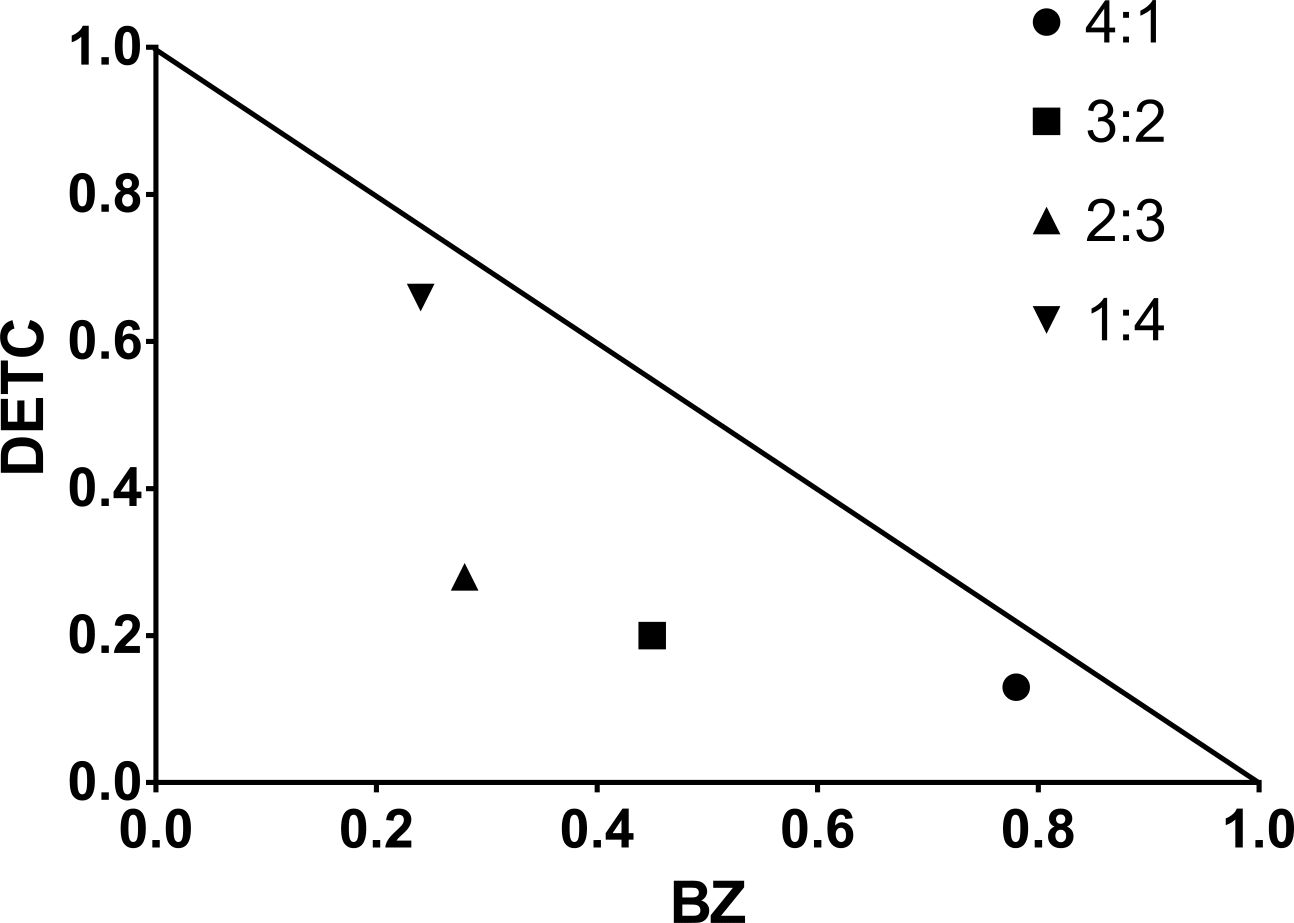
Representative isobologram demonstrating the synergistic interaction between DETC and BZ upon *T. cruzi* (Y strain) epimastigote forms, cultured for 24 h, based on IC_50_ values.

**Figure 3:**
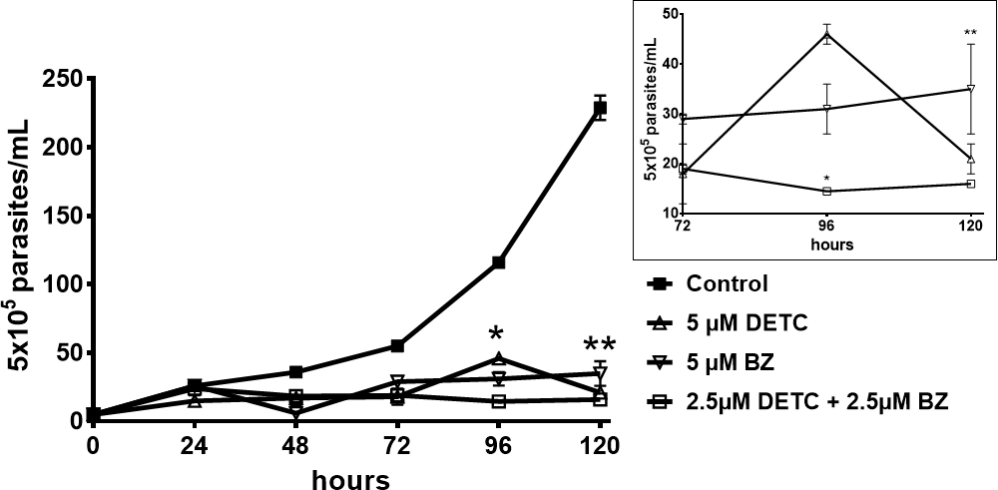
Evaluation of the *in vitro* proliferation of *T. cruzi* epimastigote forms (Y strain), challenged with DETC and BZ combined or alone. 10^5^ parasites were incubated for 5 days with 5 µM DETC, 5 µM BZ and the combination of 2.5 µM each compound. The effects of the compounds were evaluated by daily quantitation of parasites by light microscopy. Inset displays 72-120 h data in a different scale. The combined drugs effectivity was significant, (*p < 0.05; **p < 0.01), despite the reduced concentrations, as compared to the parasites treated with BZ by the 2-way ANOVA. Data represent the mean ± SD (n = 3).

**Figure 4:**
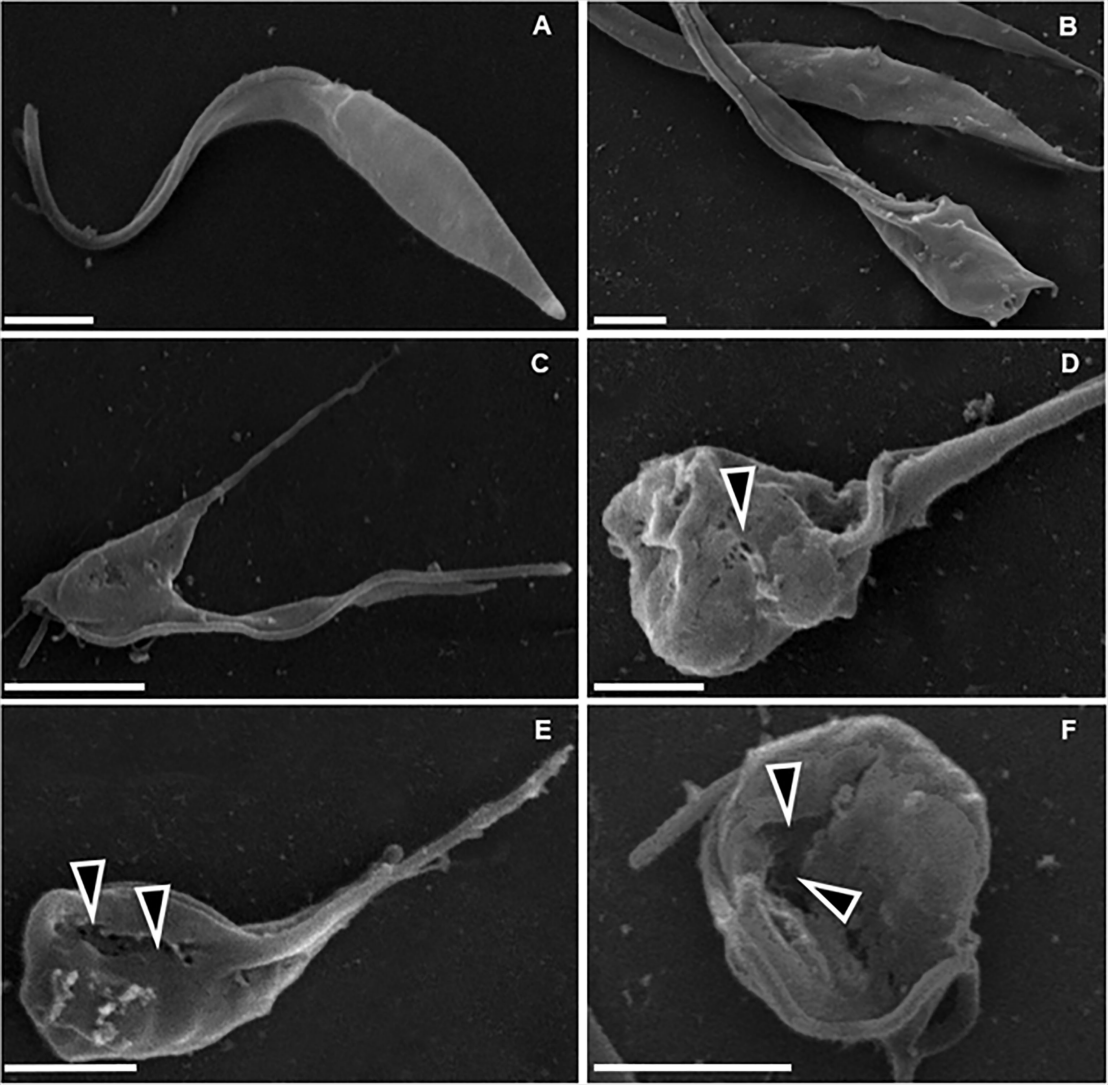
Scanning electron microscopy of *T. cruzi* epimastigote forms (Y strain). **(A)** Untreated control, showing normal parasite morphology. **B–F**) Parasites treated with 200 µM DETC for 24 h, showing cellular disorganization **(B, C)** and plasma membrane discontinuities (**D–F**, arrowheads), associated with cell body rounding and volume reduction. Bars correspond to 1 µm.

**Figure 5:**
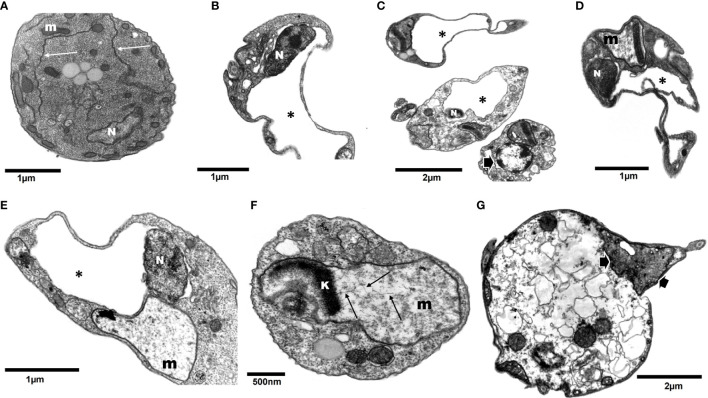
Transmission electron microscopy of *T. cruzi* epimastigotes (Y strain) incubated with 200 µM DETC for 24 h. Contrary to control cells (**A**, arrows – endoplasmic reticulum cisternae; m- mitochondria; N- nuclei), DETC treated **(B–G)** displayed remarkably enlarged endoplasmic reticulum cisternae (B–E*), often replacing an even distorting nuclei **(B, C)**. Some parasites displayed electroluscent nuclear matrix with aggregated peripheral dense chromatin (**C**, arrow). Mitochondria were enlarged and generally presented reduced electrondensity **(D–F)** and eventually presenting kinetoplast (k) DNA (kDNA) disorganized fibers (**F**- arrows). Some cells displaying condensed mitochondria presenting swollen cristae (**C, G**-arrows) were also observed. In the final stage of treatment, parasites showed loss of normal compartmentation **(G)**.

DETC inhibited dose-dependently the axenic proliferation of epimastigote forms (**Figure 1**). The IC_50_ value obtained was 1.48 µM. The BZ IC_50_ observed was 2.28 μM (**Table 1**). The selectivity indexes revealed that the combination selectivity was increased over an order of magnitude (about 13-fold), as compared to the drug of choice, BZ. Based on the IC_50_ values obtained DETC and BZ were combined at different proportions. Isobolograms were plotted to analyze the possible synergism between BZ and DETC on the trypanocidal activity. There was synergistic activity, particularly at 3:2 and 2:3 concentration ratios (**Figure 2**). Afterward, we observed that 5 µM of either BZ or DETC significantly inhibited parasite growth, whereas the combination of 2.5 µM of each compound was even more effective (**Figure 3**). In order to approach the DETC effects upon general parasite structure, we employed scanning electron microscopy. DETC-treated epimastigotes were often bizarrely shaped, eventually presenting surface discontinuities and reduced cell body volume (**Figure 4**). We used transmission electron microscopy to determine the DTEC effects on epimastigote subcellular architecture. DETC-treated parasites displayed remarkably enlarged endoplasmic reticulum (ER) cisternae as well as reduced mitochondrial electrondensity (**Figure 5**). Morphometric analysis indicate the ER lumen was enhanced over 1000-fold (not shown). Some parasites displayed disorganized kinetoplast DNA and loss of cell ultrastructural compartmentation.

**Table 1:** Trypanocidal activity, cytotoxicity, and selectivity indexes of the DETC + BZ combination after 24 h of treatment.

Combination ratio (DETC : BZ)	Cytotoxicity (peritoneal cells)	*Trypanosoma cruzi* (Y) epimastigotes	Selectivity indexes
	CC_50_ (µM)	IC_50_ (µM)	
5:0	2.09	1.48	1.41
4:1	20.06	0.67	29.94
3:2	147.00	1.48	99.32
2:3	0.58	0.30	1.93
1:4	1.03	1.68	0.61
0:5	17.54	2.28	7.69

The CC_50_ and IC_50_ values of the combination were calculated from the cytotoxicity and inhibition assay, respectively, using Alamar Blue.

**Figure 6:**
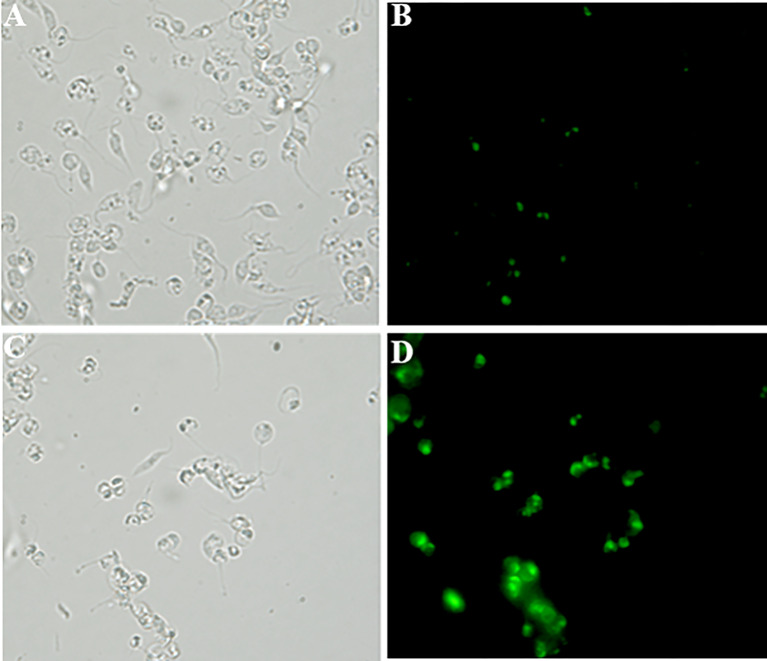
Detection of cellular reactive oxygen species (ROS) in *T. cruzi* epimastigote forms (Y strain) using the H_2_DCFDA probe accessed by fluorescence microscopy **(B, D)**. **(A, B)** - Control and **(C, D)**- 10µM DETC-treated. **(A, C)** - phase contrast images. Note intense and compartmented staining in DETC-treated parasites. Magnification - 400X.

**Figure 7:**
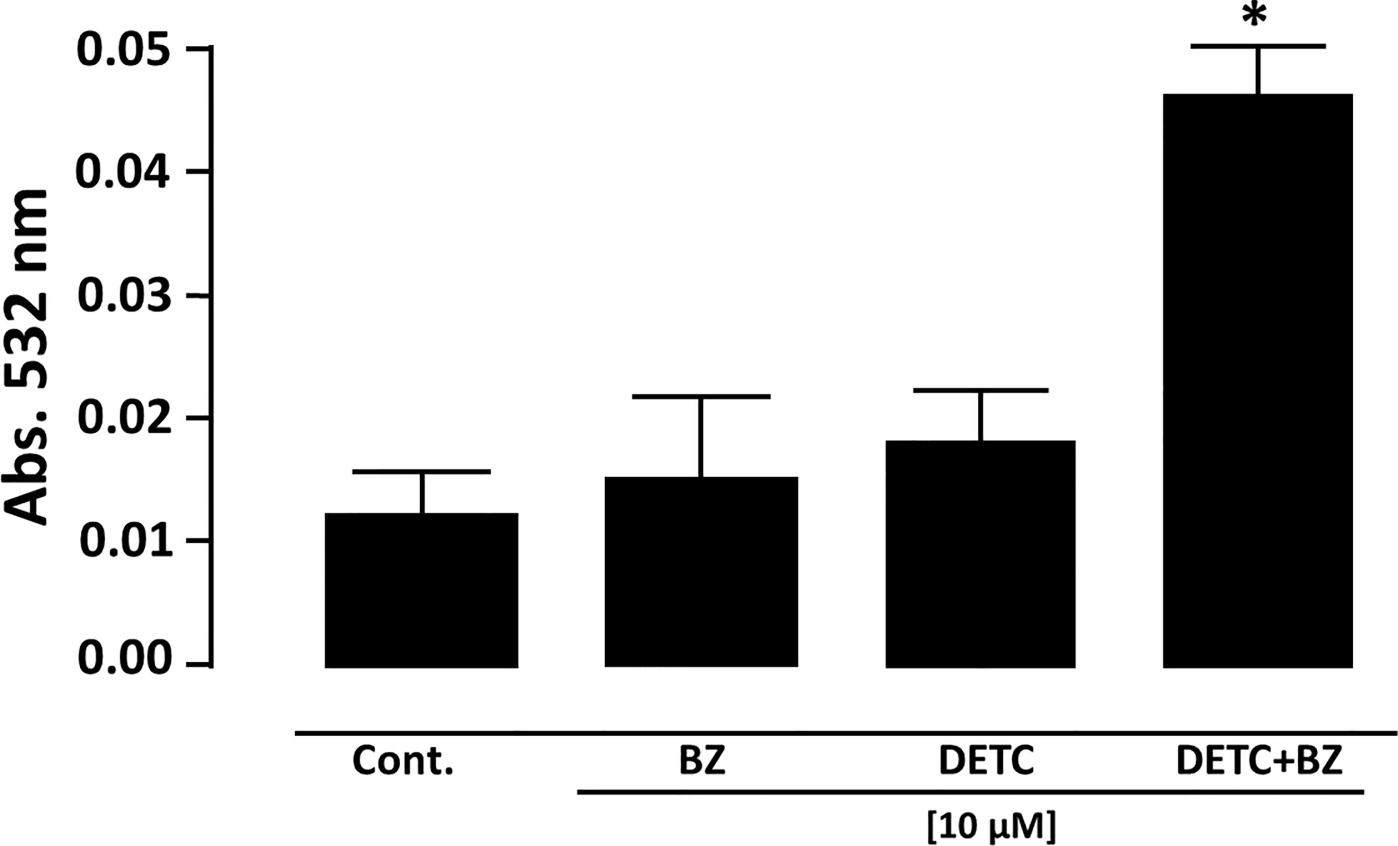
Measurement of lipid peroxidation in *T. cruzi* epimastigote forms (Y strain) by determination of thiobarbituric acid reactive substances (TBARS). Parasite cells treated with 10 µM DETC and 10 µM BZ combination for 24 h presented a significantly (*p < 0.05, ANOVA and Dunn’s post-test) increased lipoperoxidation. Bars represent mean ± SD (n = 4).

**Figure 8:**
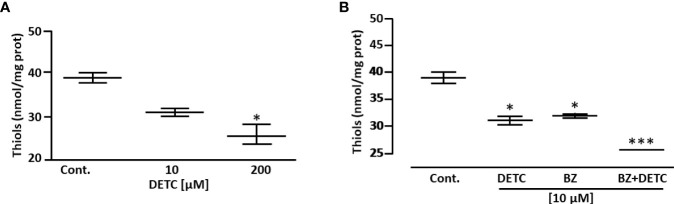
Effect of DETC and BZ on the concentration of low mol. wt. thiols of *T. cruzi* epimastigote forms (Y strain), determined colorimetrically by the Ellman reaction. DETC reduced thiol levels dose-dependently **(A)**. The DETC + BZ combination significantly potentiated thiol depletion **(B)**. Data represent mean ± SD. *p < 0.05 and ***p < 0.001 (ANOVA and Dunn’s post-test; n = 5).

In order to approach reactive oxygen species (ROS) production in DETC-treated parasites, we used fluorescent probes. Incubation with DETC remarkably enhanced H_2_DCFDA staining under fluorescence microscopy (**Figure 6**) and labeling was found in sub-cellular compartments, rather than whole cell. To evaluate the oxidative stress consequence, we measured lipoperoxidation using the TBARS assay (**Figure 7**). We observed that both BZ and DETC had little effect (*p >*0.05) on lipid peroxidation, but it was significantly (*p* < 0.05) increased by their combination. Since sulfhydryl groups are largely involved in the redox regulation in trypanosomatid parasites, we measured thiol expression using the colorimetric Ellman’s reaction DETC diminished thiol expression in the parasite (**Figure 8**). The biochemical colorimetric approach indicates that DETC produce a dose-dependent effect and that the isolated compounds reduced SH levels (*p* < 0.05), but the combination was more effective (*p* < 0.01). As DETC is well-known for its SOD inhibiting capacity, we measured SOD activity *in vitro* and *in vivo*, in murine infection, before and after DETC treatment (**Table 2**). Interestingly, the combination was more inhibitory, and the effects *in vitro* were more pronounced.

**Table 2:** Evaluation of *T. cruzi* superoxide dismutase (SOD) activity in presence of DETC *in vitro* and DSF *in vivo* isolated and combined to BZ.

Assay		SOD activity (U/mL)					
	Untreated (1% kolliphor)	BZ (100 µM)				DETC (100 µM)		BZ + DETC (50:50 µM)	
*In vitro^1^* 1h	2.975	2.821				3.056		2.103**	
*In vitro^1^* 24h	0.896	0.705				0.773*		0.734*	
	Untreated (1% kolliphor)	BZ (50 mg/kg)		DSF(100 mg/kg)		BZ + DSF (50:50 mg/kg)		BZ + DSF (50:100 mg/kg)
*In vivo^2^*	0.059	0.059		0.058		0.063		0.052

^1^ *T. cruzi* epimastigote forms (Y strain), treated or not with DETC isolated or in combination with BZ, spectrophotometrically measured.

^2^SOD activity of trypomastigote (Y strain)-infected mice treated for 3 consecutive days. * p < 0.05 and ** p < 0.01 ANOVA and Dunn’s post-test (n = 3).

**Figure 9:**
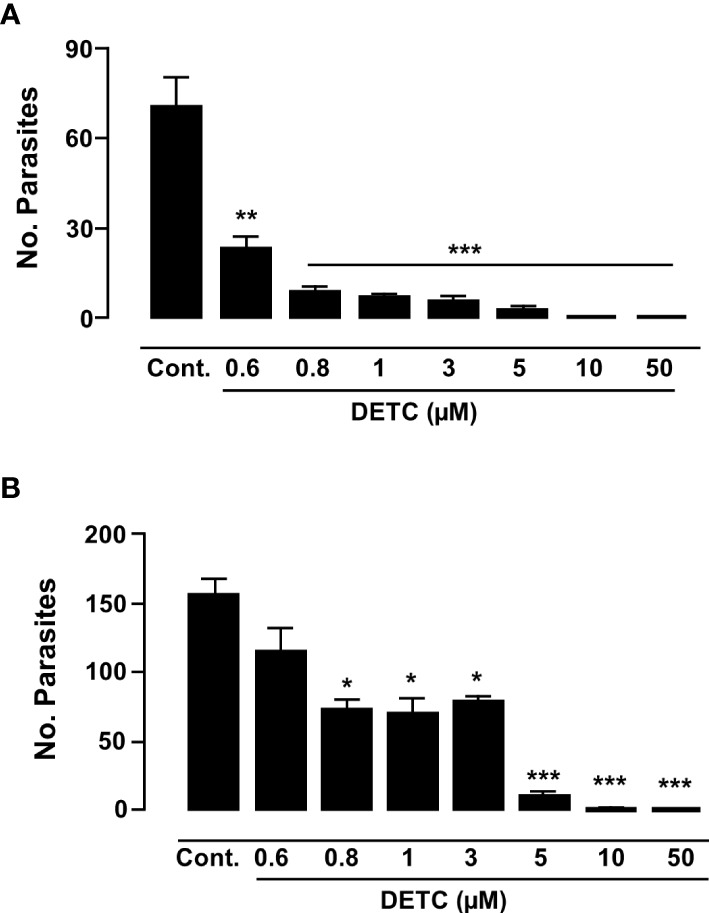
Inhibitory effect of DETC on the proliferation of *T. cruzi* amastigote forms in Y **(A)** and Colombiana **(B)** strains, after incubations of 120 h and 24 h, respectively. * *p* < 0.05, **p < 0.01 and *** indicates *p* < 0.001 ANOVA and Dunn’s post-test. Bars represent the mean ± SD (n=3).

As the Y strain is sensitive to BZ, we decided to test the DETC susceptibility of axenic amastigotes of both Y and Colombiana strains. Although the Colombiana strain, naturally resistant to BZ, was less sensitive to DETC at 0.8-3 µM (*p* < 0.05), the effects were highly significant (*p* < 0.01) at 5 µM. Both strains showed significantly decreased parasite survival in a dose-dependent manner (**Figure 9**).

**Figure 10:**
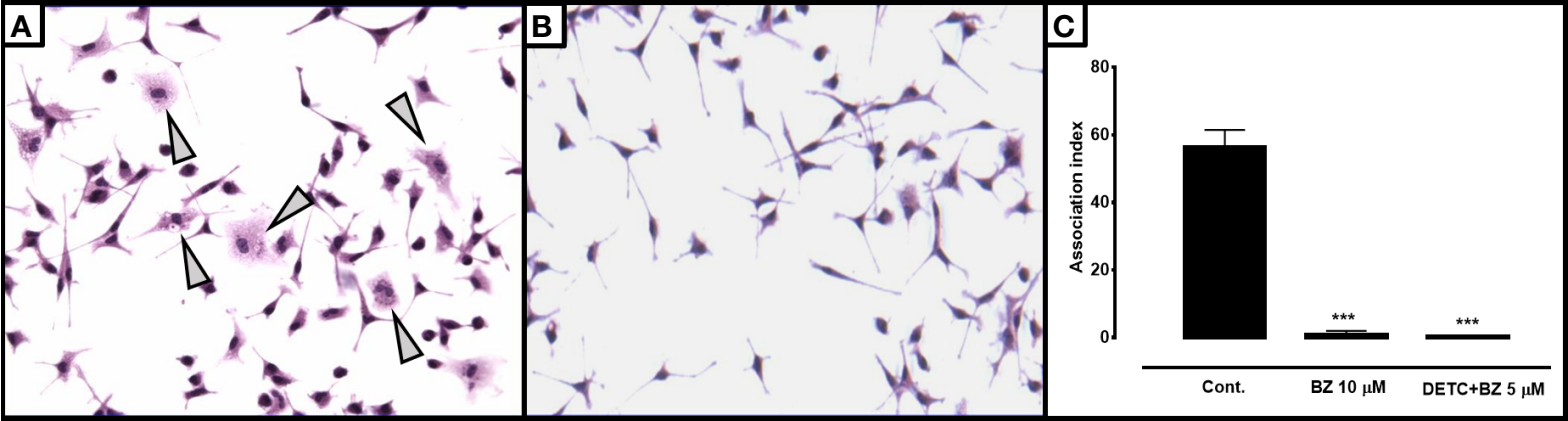
Effect of DETC alone or combined with BZ on the *T. cruzi*-macrophage interaction. BALB/c mice peritoneal macrophages were infected with Colombiana strain bloodstream trypomastigote forms 1:10 phagocyte/parasite ratio. Control cultures **(A)** showing several parasitized macrophages as well as larger number of intracellular forms per phagocyte (arrowheads) than cells treated with the combination 5 µM DETC + 5 µM BZ **(B)**. Magnification 200X. The association index in cultures treated with 10 µM BZ or the 5 µM DETC + 5 µM BZ combination after 24 hours of incubation **(C)** was significantly (***p < 0.001, ANOVA and Dunn’s post-test) reduced. A slightly greater inhibition was observed in cultures incubated with the combination, despite the reduced concentrations. Bars represent mean ± SD.

In order to test the effects of the combination in intracellular parasites, we infected murine macrophages with blood trypomastigotes in the presence or in the absence of DETC (**Figure 10**). We noticed that 10 µM DETC remarkably reduced the monolayer parasite load, and a DETC-BZ combination at 5 µM concentration was equally or even more effective.

**Figure 11:**
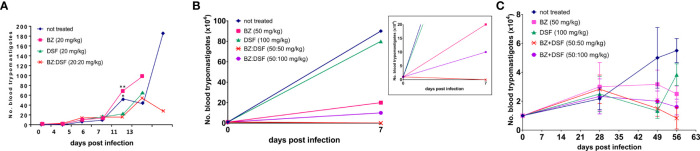
Parasitemia of Swiss Webster mice infected with *T. cruzi* blood trypomastigotes, Y strain **(A, B)** and Colombiana **(C)**. The animals were infected with 10^4^ blood trypomastigotes/mL, strain Y, treated for 60 days with vehicle; low doses of BZ (20 mg/kg); DETC (20 mg/kg) or a combination of BZ + DETC (20:20 mg/kg), orally. *p < 0.05 and **p < 0.01 ANOVA test and Dunn’s post-test **(A)**. Parasites treated with vehicle; BZ (50 mg/kg); DSF (50 mg/kg); combination of BZ + DSF (50:50 mg/kg); and BZ + DSF (50:100 mg/kg), administered for 10 consecutive days **(B**, **C**, *p* > 0.05**)**. Insert shows data in a different scale. The parasites were counted daily **(A)** or weekly **(B, C)** on Neubauer chambers and the graphs were plotted in GraphPad Prism, 7.

**Figure 12:**
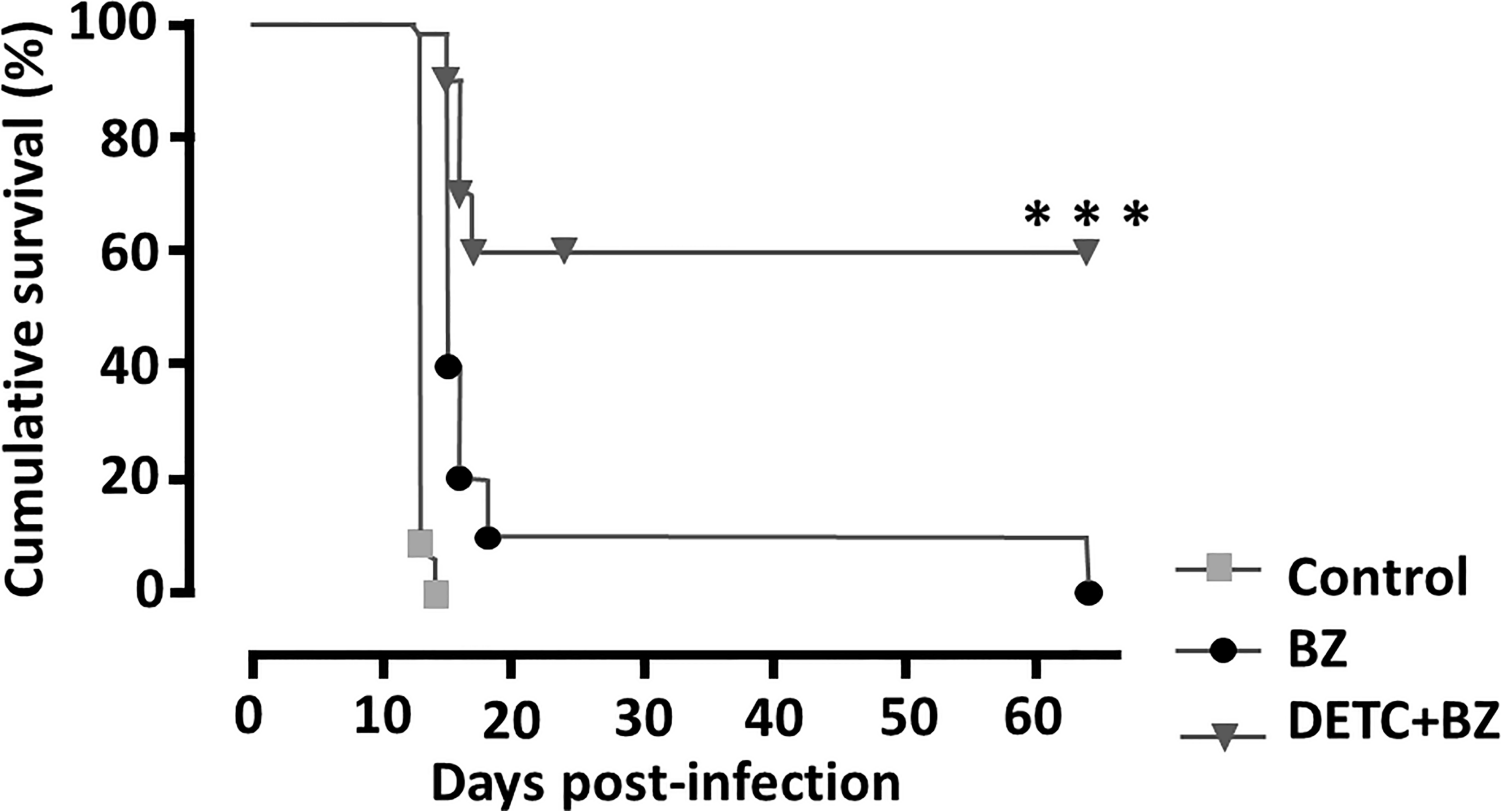
Monitoring the cumulative survival of mice infected intraperitoneally with 10^4^ *T. cruzi* blood trypomastigote/mL (Y strain) of and treated with 10 mg/kg/day of DETC and BZ alone or in combination, p.o. daily for 60 consecutive days. Note that animals treated with the combination had a survival rate of 60%, whereas the group treated with BZ was 10% and the control group died on the 14^th^ day. ***p < 0.001 ANOVA and Tukey post-test.

**Figure 13:**
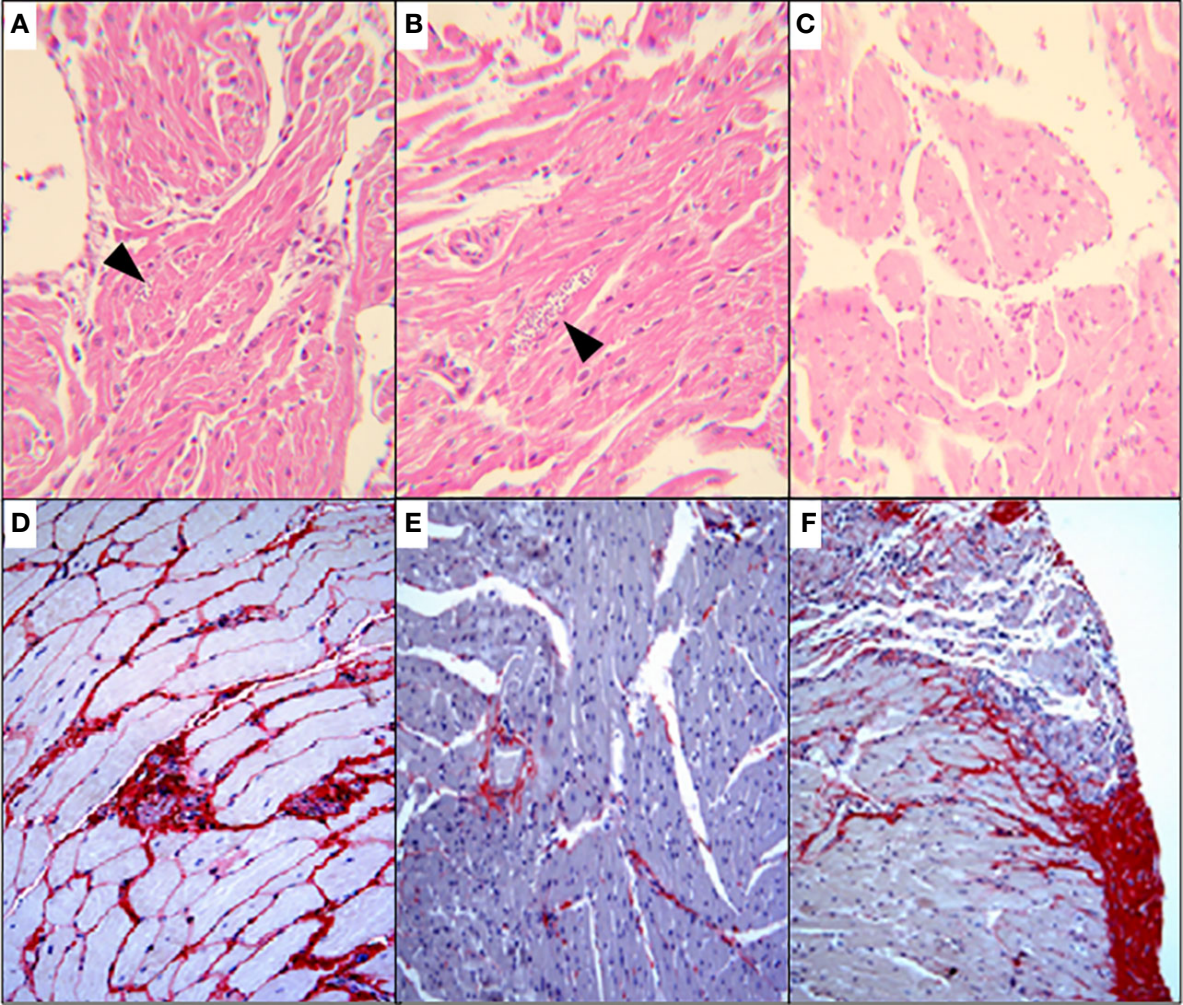
Histopathological analysis (H&E) of murine myocardium *T. cruzi* (Y strain)-infected mice, treated for 60 consecutive days, p.o. with vehicle **(A)**; 10 mg/kg/d BZ-treated and **(B)** treated with the of 10 mg/kg/d DETC combined to 10 mg/kg/d BZ **(C)**. Amastigote nests were observed in A and B (arrowheads). Skeletal muscle of mice infected with Colombiana strain, for 100* d* and treated for 30 consecutive days orally with 20 mg/kg/d BZ **(D)**, 20 mg/kg/d DSF **(E)** or combination of 10 mg/kg/d each **(F)**. Picro Sirius red staining was employed to demonstrate fibrosis. The combination using lowered concentrations was associated with reduced fibrosis **(F)**. Magnification: 200X.

**Figure 14:**
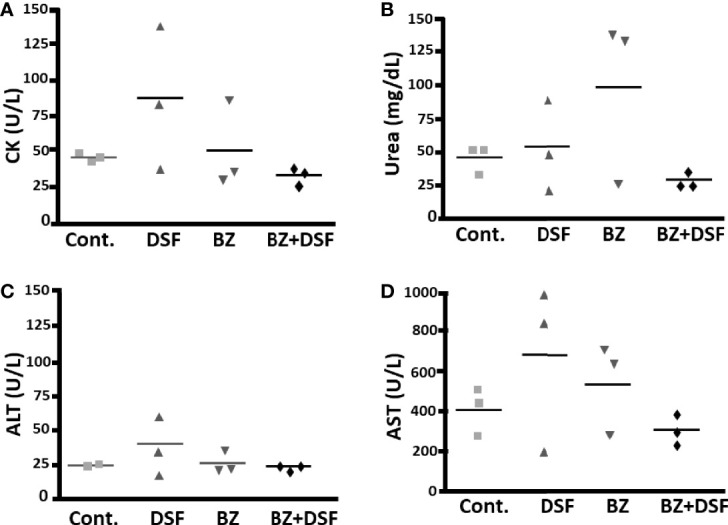
Systemic toxicity of mice treated with DSF and BZ, alone and in combination, by determining blood levels of creatine kinase **(A)**, urea **(B)**, alanine aminotransferase **(C)** and aspartate aminotransferase **(D)**; after 20 mg/kg BZ or DSF and the 10 mg/kg/d BZ + 10 mg/kg/d DSF. Contrary to isolated drugs, the combination, employing lowered dosages, did not increase plasma levels.

To approach the activity of the combination *in vivo*, we tested the murine infection for 30-60 days. The parasitemia of animals infected with both Y and Colombiana strains was reduced by the BZ-DSF combination (**Figure 11**) as compared to BZ low concentrations (20/50 mg/kg). The cumulative survival of the animas treated with the 10 mg/kg/day (each) DETC-BZ combination was increased by circa 6-fold (*p* < 0.01) as compared to BZ alone (**Figure 12**). We used histopathology to evaluate the murine infection. Contrary to animals treated with low BZ concentration (10 mg/kg/d) the ones incubated with the combination (10 mg/kg/d BZ + 10 mg/kg/d DETC) showed no amastigote nests in myocardium (**Figure 13**). The Sirus red staining was employed to assess tissue fibrosis. Animals treated with the combination displayed less, and focal fibrosis, whereas mice treated with BZ alone showed intense fibrosis staining. To evaluate the systemic toxicity of the treatments we measured the plasma levels of ALT, AST, CK, and urea. Measurements demonstrated (**Figure 14**) that the combination at 10 mg/kg/day (each) was not as toxic than the isolated drugs at 20 mg/kg/day.

## Discussion

CD remains a major public health problem, as most of the infected people and domestic animals such as dogs, important reservoir hosts (Enriquez et al., 2014; Castillo-Neyra et al., 2015), are not treated or diagnosed (Dias et al., 2016). Since both drugs employed in CD chemotherapy are remarkably toxic (Castro and Diaz de Toranzo, 1988; Castro et al., 2006), therapy is frequently associated with severe adverse reactions, often causing treatment suspension (Levi et al., 1996; Olivera et al., 2017; Guggenbühl Noller et al., 2020; Pérez-Molina et al., 2021). Development of new safe and low-cost therapeutic alternatives is urgently required. Drug repositioning and combinations comprise valuable tools for overcoming CD monotherapy limitations (e.g., Aguilera et al., 2019).

Both DETC and DSF express antiparasitic activities upon *T. cruzi* (Lane et al., 1996; de Freitas Oliveira et al., 2021; de Freitas Oliveira et al., 2022) and *Leishmania* (Khouri et al., 2010; Celes et al., 2016; Mazur et al., 2019). In macrophage cultures, DETC caused destruction of intracellular amastigotes with little effect on macrophage spreading pattern. Similarly, in the murine infection, the combination reduced parasitemia and significantly (*p <* 0.001) enhanced animal survival by circa 6-fold. Synergistic combinations can enhance selectivity (Zimmermann et al., 2007; Lehár et al., 2009a; Lehár et al., 2009b). In fact, the BZ + DETC combination was synergistic *in vitro* and *in vivo* and produced over 10-fold selectivity index increase. Such synergistic activities may be caused by the prooxidant effects of BZ (Pedrosa et al., 2001; Rigalli et al., 2016), associated with the antagonism of antioxidant defenses of DSF and that can allow dosing reduction (Bustamante et al., 2014), presumably leading to decreased adverse reactions. Besides, DSF and DETC were shown to reduce adverse reactions of other drugs (*vide supra*). Here we observed that reduced combined drug concentrations did not impair effectivity. Therefore, such combinations may lead to the development of safe and effective medications.

Since one of the major problems in CD therapy is the prompt rise of resistance, we tested the combination in axenic amastigotes as well as in infected macrophages, not only of the Y strain but also of the Colombiana one, highly drug-resistant (Andrade et al., 1985). Interestingly, both strains were sensitive to the thiocarbamates at low µM range. Drug combinations were employed for overcoming drug resistance rising but were not effective in leishmaniasis (García-Hernández et al., 2012) or malaria (Wongsrichanalai et al., 2002) and drug combinations were not effective upon drug-resistant *T. cruzi* (Bustamante et al., 2014).

CD treatment is confronted with growing frequencies of refractory cases. Over 60% of *T. cruzi* strains isolated in Colombia display some degree of BZ resistance (Mejía-Jaramillo et al., 2012) and strains isolated in the Brazilian Amazon show natural resistance to this drug (Teston et al., 2013). The rise of readily acquired (Neal and van Bueren, 1988; Mejia et al., 2012) or natural resistance (Zingales, 2018) significantly limit the therapeutic success of CD. Thus, a major relevance of the DSF + BZ stems from the potential revert drug resistance phenotypes, by inhibiting both activity (Loo et al., 2004; Sauna et al., 2004) and expression (Loo and Clarke, 2000) of p-glycoproteins, which are involved in *T. cruzi* drug resistance (Campos et al., 2013).

Besides ABC cassette pumps (Zingales et al., 2015), natural resistance may rely on aldo-keto reductase and alcohol dehydrogenase (González et al., 2017). In this regard, it is important to notice that DSF inhibits alcohol dehydrogenase activity (Carper et al., 1987) and decreases the expression of aldo-keto reductase accessed by proteomic analysis (Yoshino et al., 2020). In addition, glutathione (GSH) forms conjugates with different drugs that are extruded by ABC transporters (Jedlitschky et al., 1994; Loe et al., 1998), so GSH and trypanothione (TSH) are implicated in detoxication of both NFX and BZ (Repetto et al., 1996). DSF can act as a glutathionylation agent (Hirschenson and Mailloux, 2021). Similarly, DETC derivatives form conjugates with GSH *via* carbamoylation (Ningaraj et al., 1998) and this reaction inhibits glutathione reductase (Miller and Blakely, 1992). *T. cruzi* ABC proteins externalize thiol-drug conjugates (da Costa et al., 2018) and inhibition of GSH and so TSH synthesis by buthionine sulfoximine (Maya et al., 2004; Faúndez et al., 2005; Vázquez et al., 2017) augments the effectivity of trypanocidal agents BZ and nifurtimox (Faúndez et al., 2005; Faúndez et al., 2008). Interestingly, DSF/DETC can diminish reduced GSH levels (Strömme, 1963; Ohno et al., 1990; Nagendra et al., 1994; Mittal et al., 2014) and GSH/TSH depletion can trigger *T. cruzi* programmed cell death (Piacenza et al., 2007). Here we observed reduced low mol. wt. thiol levels in DETC-treated *T. cruzi*, by colorimetric and cytometric approaches, presumably by the formation of DS/DETC thiol-mediated conjugates with GSH (Jin et al., 1994). Similarly DSF reducing GSH levels, potentiate antiparasitic activity in malaria experimental models (De Jongh, 1953a; De Jongh, 1953b; Deharo et al., 2003). Therefore, the use DSF may overcome *T. cruzi* drug-resistance *via* different mechanisms.

The ultrastructural analysis performed here revealed surface membrane discontinuities, which may be explained by lipid peroxidation, corroborated by TBA determination, and the cell surface damage may lead to loss of cytoplasmic content culminating in necrosis and cell collapse observed by SEM. The analysis by TEM revealed remarkable damage on parasite ER and mitochondria, redox-active organelles. Oxidative stress induces ER stress (Liu et al., 2019; Esmaeili et al., 2022) and DSF triggers ROS-dependent ER stress (Shah O’Brien et al., 2019). The ER stress is related to the mitochondrial one (Wang et al., 2016; Kim et al., 2022; Yuan et al., 2022) and the latter can be promoted by the DETC-mediated SOD inhibition as well as by the reductions in GSH levels.

DSF causes ER stress associated with ROS production (Shah O’Brien et al., 2019). ER stress may cause the organelle cisternae dilation (Montalbano et al., 2013), which are not as conspicuous as the remarkable enlargement observed in *T. cruzi* here, *i.e.*, up to over 1000-fold, revealed by ultrastructural morphometry (not shown) and much higher than observed on mammalian cells. Such difference may be explained at least in part by a selective mechanism of action. It is noteworthy that ER stress was shown to be implicated in the CD cardiomyopathy in a murine model (Ayyappan et al., 2019). DETC can enhance ER stress in a kidney model (Kang et al., 2020), whereas its derivative DETC-MeSO (S-methyl-N, N-diethylthiocarbamate sulfoxide) can decrease ER stress markers in a stroke rat model (Mohammad-Gharibani et al., 2014).

The ubiquitin system comprises therapy target in diverse disorders (Wertz and Wang, 2019), and proteasome can be exploited as chemotherapy target for parasitic protozoa (Cromm and Crews, 2017; Xie et al., 2019). Parasite COP9 signalosomes comprise potential targets for chemotherapy (Ghosh et al., 2020). Interestingly DSF inhibits the ubiquitin-proteasome system (Cvek and Dvorak, 2008; Kona et al., 2011). The ER stress-associated cisternae dilation can be detected by EM (Riggs et al., 2005; Akiyama et al., 2009), so we employed the ultrastructural approach to help elucidating antiparasitic agents’ mechanisms of action (Vannier-Santos and De Castro, 2009). The inhibition of proteasome function causes the accumulation of precursor proteins within ER cisternae (Hoefer and Groettrup, 2021) and accumulation of misfolded proteins may cause ER dilation (Sun et al., 2014) and trigger oxidation-reduction futile cycles producing H_2_O_2_ and depleting reduced GSH pools in the ER (Malhotra et al., 2008).

The *T. cruzi* intracellular Ca^2+^ homeostasis is an important therapeutic target (Benaim et al., 2020) and calcium can be transferred from the ER to mitochondria during stress *via* mitochondria associated membranes (MAM), also termed ER–mitochondria encounter structure (ERMES), and the Ca^2+^ overload may be implicated in mitochondrial damage (Mohsin et al., 2020). We have previously described the ER-mitochondria connection in *Leishmania braziliensis* treated with histone deacetylase (HDAC) inhibitors, including the nuclear envelope in the trypanosomatid parasite  (Ângelo de Souza et al., 2020). HDAC inhibitors cause ER stress (Chen et al., 2017) and lead to the formation of mitochondria-ER tight binding, associated with ER cisternae enlargement, but it was much less pronounced than reported here. A similar mitochondria-ER association was detected here (**Figure 5E**), but since both compartments were remarkably enlarged, an eventual connection interpretation could be misleading.

Like the ER alterations, the DETC mitochondrial effects observed on the parasite cells were not detected on the mammalian cells, as previously reported in *Leishmania*-infected macrophage (Khouri et al., 2010). Remarkably enlarged mitochondria with washed out matrix were previously observed in both *T. cruzi* (Menezes et al., 2006) and *Leishmania amazonensis* (Vannier-Santos et al., 2008) exposed to 1,4-diamino-2-butanone a biocide that impairs the *T. cruzi* redox homeostasis (Soares et al., 2012) and induces lipoperoxidation (Menezes et al., 2006; Vannier-Santos et al., 2008). This putrescine analogue can inhibit polyamine transport and biosynthesis, affecting numerous parasite cell functions (Vannier-Santos and Suarez-Fontes, 2017), therefore involving different pharmaceutical targets (Roberts and Ullman, 2017). Polyamines play a pivotal role in trypanosomatid parasites, including the antioxidant protection from lipid peroxidation (Hernández et al., 2006). Also, this polycation metabolism is important for the antioxidant role of the spermidine-GSH adduct Trypanothione (N1, N8-Bis(glutathionyl)spermidine, TSH). TSH metabolism and SOD are drug targets for *T. cruzi* infection (Beltran-Hortelano et al., 2017; Piñeyro et al., 2021). Both NFX and BZ interfere with TSH metabolism (Maya et al., 1997; Maya et al., 2007), and TSH confers *T. cruzi* resistance to BZ and NFX (Maya et al., 2004; Faúndez et al., 2005; Mesías et al., 2019). TSH and tryparedoxin take part in BZ-resistance (González-Chávez et al., 2019) and the enzyme comprises a drug target (González-Chávez et al., 2015).

The fluorescent probe H_2_DCFDA labeling indicates the ROS production and the oxidative stress triggering may be due both to SOD inhibition by DETC (Heikkila et al., 1976; Heikkila et al., 1978) as well as to lowering reduced glutathione levels (vide supra). The DSF-mediated glutathionylation enhances the O_2_^●-^ and H_2_O_2_ production in mitochondria (Hirschenson and Mailloux, 2021) and SOD inhibition was previously shown to reduce *T. cruzi* parasitemia in murine infection (Olmo et al., 2015). The SOD inhibition observed here was significant on the parasite *in vitro*, but not *in vivo*, again possibly indicating a selective mechanism. Interestingly, the *T. brucei* SOD gene *sodb1* deletion increases sensitivity to NFX and BZ (Prathalingham et al., 2007) and SOD inhibition enhances NFX antitumoral activity (Koto et al., 2011). In this regard, the DSF and DETC strongly inhibit *T. cruzi* SOD (Giulivi et al., 1988), and is trypanocidal (de Freitas Oliveira et al., 2021), also exerting leishmanicidal effects (Khouri et al., 2010). Interestingly, the *T. cruzi* Fe-SOD is found within parasite mitochondria, preventing programmed cell death (Piacenza et al., 2007). The immunofluorescence labeling of TcSOD in the parasite kinetoplast is relevant since it can preclude DNA fragmentation (Piacenza et al., 2007). In the TEM images shown here, we observed DETC-treated parasites with kDNA disorganization as well as mitochondrial damage. In this regard, arginase inhibition, causing redox imbalance, lead to kDNA disorganization in *Leishmania* (Cruz et al., 2013). SOD downmodulation may pose a dual advantage in CD therapy, since the enzymes are involved in intracellular survival, as scape mechanism from macrophage production of superoxide (Martínez et al., 2019), but also drug resistance (Nogueira et al., 2006; Campos et al., 2014; Campos et al., 2017). In this regard, the Sirtuin TcSir2rp3 induces the overexpressing TcFeSOD-A activities increasing *T. cruzi* resistance to BZ and NFX (dos Santos Moura et al., 2021).

Thiocarbamates chelate copper and trigger its accumulation and lipid peroxidation (Tonkin et al., 2004; Valentine et al., 2009; Viquez et al., 2009). Besides, copper-containing nanoparticles trigger oxidative stress and ER stress (Liu et al., 2021). Copper chelated by DSF induces cancer cells apoptosis in a ROS and mitochondria-dependent manner (Ren et al., 2021).

Ferroptosis is a programed cell death mechanism (Stockwell et al., 2017) characterized by mitochondria with enhanced matrix electron density and swollen cristae (Dixon et al., 2012). Such alterations were observed by TEM here, so this mechanism may be triggered by DSF. It should be kept in mind that different cell death mechanisms such as necrosis, apoptosis, and autophagy may be triggered simultaneously (dos Anjos et al., 2016). Interestingly, in a murine model of sepsis, the induction of pyroptosis and ferroptosis is associated with downregulation of the mitochondrial aldehyde dehydrogenase (Cao et al., 2022) and since DSF and DETC inhibit this enzyme (Deitrich and Erwin, 1971) may also trigger ferroptosis on parasite cells.

Inhibition of cystine uptake, employed in GSH (Badgley et al., 2020) synthesis, transport causes ER stress associated with ferroptosis in cancer cells (Dixon et al., 2014) and tryparedoxin peroxidase-deficient *Trypanosoma brucei* parasites undergo ferroptosis (Bogacz and Krauth-Siegel, 2018). Mitochondria are involved in cysteine-deprivation leading to ferroptosis (Gao et al., 2019).

The drug combination using DSF offers important advantages, which may be both economical (Cvek, 2012; Soave et al., 2016) and pharmacological, since this compound protects normal cells (Jia and Huang, 2021), can diminish the toxicity of different drugs such as cis-diamminedichloroplatinum (Wysor et al., 1982), including myocardial protection (Sonawane et al., 2018) and *circa* 30% of the CD patients develop heart disease, characterized by arrhythmias among other cardiac manifestations (Saraiva et al., 2021). It is worth mentioning that DSF can suppress cardiac arrhythmogenesis (Sander et al., 2021). Nevertheless, DETC and DSF were reported to cause neuropathies (Worner, 1982; Frisoni and Di Monda, 1989). In addition, BZ and NFX may cause neuropsychiatric reactions (Jackson et al., 2020). In this regard, it is noteworthy that DSF and its derivatives were also reported to act as neuroprotective agents (Zhao et al., 2000; Ningaraj et al., 2001; Libeu et al., 2012; Mohammad-Gharibani et al., 2014; Prentice et al., 2015). The eventual neurotoxic activity may be due at least in part to high dosages used in the past (Gessner and Gessner, 1992) and oxidative stress produced by copper accumulation leading to lipid peroxidation (Tonkin et al., 2004), although DSF may exert antioxidant effects (Kyle et al., 1989). This property is relevant since both BZ (Pedrosa et al., 2001; Rigalli et al., 2016) and CD (de Oliveira et al., 2007; Gupta et al., 2009) are associated with oxidative stress.

Histopathological samples revealed no amastigote nests detected in tissues of animals treated with low doses of the DSF-BZ combination, which showed mild inflammatory infiltrates and did not display significant fibrosis as assessed a by the picrosirius red staining. CD is an inflammatory infection (Talvani and Teixeira, 2011) associated with fibrosis and pyroptosis (Cerbán et al., 2020). The *T. cruzi* infection leads to ROS formation in the mitochondria, triggering PARP-1 and NF-kappaB activation, culminating in the production of pro-inflammatory cytokines such as Il-12 and IFN-γ (Ba et al., 2010), involved in cardiomyopathy pathophysiology (Cunha-Neto et al., 1998). In this regard, DSF is anti-inflammatory (Kanai et al., 2011), of potential use for inflammatory disorders (Guo et al., 2022), inhibiting oxidative stress and inflammasome activation, preventing cardiac damage (Wei et al., 2022), suppressing fibrosis (Zhang et al., 2021) and pyroptosis (Hu et al., 2020; Li et al., 2022; Zhou et al., 2022). The immune response to *T. cruzi* infection associated with myocardial fibrosis involves TGF-β (Chaves et al., 2019), and this cytokine comprises a therapeutic target in the CD cardiac damage (Waghabi et al., 2022). In this regard, DSF can inhibit TGF-β receptor signal transduction (Jiang et al., 2021).

Systemic toxicity assays based on the measurement of plasmatic CK, AST, ALT, and urea indicate decreased or insignificant toxicity. DSF was shown to reduce toxicity of several drugs such as cis-diamminedichloroplatinum, a property termed “disulfiram rescue” for saving the treated organism (Wysor et al., 1982; Roemeling et al., 1986). DETC is also considered a rescue agent (Gandara et al., 1990).

Because of promising *in vitro* and *in vivo* results, we are starting a clinical trial with the DSF-BZ combination (Saraiva et al., 2021), comprising a *bona fide* translational approach. Taken together, the present data and the cytoprotective capacity of DSF/DETC as well as the resistance reversion potential, such a combination may be safe and effective, even in refractory CD cases.

## Data availability statement

The raw data supporting the conclusions of this article will be made available by the authors, without undue reservation.

## Ethics statement

The animal study was reviewed and approved by Comissão de Ética no Uso de Animais do Instituto Oswaldo Cruz/Fiocruz. lic. no. L012/2020.

## Author contributions

JA-S, DM, JF, MA, and DV-d-S: experiment execution; RS, AV, SP, AS-F: Data interpretation, discussion and translational research planning; SA, Histopathological analysis; MV-S: Project conception, study planning, funding obtaining, student supervision, microscopy interpretation; discussion. All authors contributed to the article and approved the submitted version.

## Funding

Conselho Nacional de Desenvolvimento Científico e Tecnológico (CNPq), grant number 314717/2020 and Fundação Carlos Chagas Filho de Amparo à Pesquisa do Estado do Rio de Janeiro (FAPERJ) grant number 260475/2021, 259286/2021, provide most of the scholarships of the team as well as consumables used in the experiments. Oswaldo Cruz Institute (IOC)/Oswaldo Cruz Fundation (Fiocruz) provides the general infrastructure and supports the open access publication fees.

## Acknowledgments

The authors acknowledge the valuable partnership of Farmanguinhos and INI/Fiocruz as well as the contributions FIOCRUZ technological platforms. To Dr. Marcus Fernandes Oliveira for valuable discussions and critical reading of the manuscript.

## Conflict of interest

The authors declare that the research was conducted in the absence of any commercial or financial relationships that could be construed as a potential conflict of interest.

## Publisher’s note

All claims expressed in this article are solely those of the authors and do not necessarily represent those of their affiliated organizations, or those of the publisher, the editors and the reviewers. Any product that may be evaluated in this article, or claim that may be made by its manufacturer, is not guaranteed or endorsed by the publisher.
